# Entomopathogenic Nematode Species Vary in Their Behavior and Virulence in Response to Cardiac Glycosides Within and Around Insect Hosts

**DOI:** 10.1007/s10886-025-01563-9

**Published:** 2025-01-27

**Authors:** Perla Achi, Preston Christensen, Victoria Iglesias, Cullen McCarthy, Robert Pena, Lanie Bavier, Connor Goldy, Anurag A. Agrawal, Simon C. Groen, Adler R. Dillman

**Affiliations:** 1 Department of Nematology, University of California Riverside, Riverside, CA USA; 2 Department of Ecology and Evolutionary Biology, Cornell University, Ithaca, NY USA; 3 Department of Entomology, Cornell University, Ithaca, NY USA; 4 Center for Infectious Disease and Vector Research, Institute for Integrative Genome Biology, University of California Riverside, Riverside, CA USA; 5 Department of Botany & Plant Sciences, University of California Riverside, Riverside, CA USA

**Keywords:** Cardizac glycosides, Cardenolides, EPNs, *Steinernema carpocapsae*, Parasitism, Milkweed

## Abstract

Plants produce defensive toxins to deter herbivores. In response, some specialized herbivores evolved resistance and even the capacity to sequester toxins, affecting interactions at higher trophic levels. Here, we test the hypothesis that potential natural enemies of specialized herbivores are differentially affected by plant toxins depending on their level of adaptation to the plant-herbivore system. We focus on toxic cardiac glycosides (CGs) from milkweeds (*Asclepias* spp.), which inhibit animal Na^+^/K^+^-ATPases, and two CG-resistant insects, the large milkweed bug *Oncopeltus fasciatus* and a CRISPR-edited *Drosophila melanogaster*. Both have CG-resistant Na^+^/K^+^-ATPases through a set of key amino acid substitutions, which facilitate CG sequestration. We conducted infection experiments with entomopathogenic nematodes (*Steinernema carpocapsae*, *S. feltiae*, and *S. hermaphroditum*) as natural enemies on host insects containing mixtures of milkweed-derived CGs or purified CGs (ouabain, digoxin, and digitoxin) that vary in toxicity. The nematode *S. carpocapsae* is known to occur in soil near milkweed plants and naturally has several of the same Na^+^/K^+^-ATPase substitutions as the milkweed bug *O. fasciatus* and our *Drosophila* mutant. This nematode not only exhibited higher fecundity in hosts that carried CGs relative to the other nematode species (which have sensitive Na^+^/K^+^-ATPases), but also showed attraction to mixtures of CGs in milkweed root extracts and to purified ouabain when tested on agar plates. A coiling phenotype, which is a symptom of neurotoxicity, was observed more frequently in *S. feltiae* and *S. hermaphroditum* upon exposure to milkweed root extracts than in *S. carpocapsae*. Nematode behavior was further tested in sand, and while attraction to CGs was found for *S. carpocapsae*, nematodes of the other species tended to migrate away from milkweed root chemicals. Thus, *S. carpocapsae* can tolerate CGs and may use these as chemical cues to locate insect hosts that live on or around milkweed plants.

**Supplementary Information:**

The online version contains supplementary material available at 10.1007/s10886-025-01563-9.

## Introduction

Multi-trophic interactions play important roles in shaping complex food webs throughout ecosystems. Many of these interactions occur around plants, which are at the base of food webs as members of the first trophic level. Plants are attacked by numerous herbivorous insects and defend themselves through mechanisms such as toxin production and the release of chemical cues from their roots and shoots that may recruit natural enemies of these insects (Gols 2014). In response, herbivorous insects have evolved mechanisms to tolerate plant toxins and, in some cases, even to sequester them for use against their natural enemies. These mechanisms include, but are not limited to, metabolic detoxification, target site insensitivity (TSI) via amino acid substitutions in the molecular targets of toxins, and/or clearing of toxins by ABC transporters and organic anion transport polypeptides (OATPs) expressed in the digestive system, the blood-brain barrier, and the Malpighian tubules (Groen et al. 2017; Groen and Whiteman 2022).

When an herbivorous species evolves sequestration of toxins from its host plants, this has the potential to drive evolutionary changes across multiple trophic levels: while natural enemies do not feed directly on the producers of the toxins (the plants), they nevertheless encounter them upon attacking toxin-sequestering herbivorous hosts or prey. As an illustration of this concept, previous studies have shown that nematodes in regions with the western corn rootworm, which sequesters toxic benzoxazinoids from maize, have evolved behavioral and metabolic resistance to these toxins in their host (Zhang et al. 2019).

Toxins provide self-defense across a vast variety of organisms. Among distinct classes of toxins, cardiac glycosides (CGs) represent a prevalent group produced by a diverse array of species, including plants such as milkweeds, foxglove, and oleander; insects such as certain chrysoline beetles and fireflies; and amphibians such as the cane toad (Agrawal et al. 2012; Mohammadi et al. 2022). Several other species, while not capable of synthesizing CGs themselves, have evolved mechanisms that allow CG resistance, facilitating CG sequestration. Examples of species that sequester CGs for self-defense are the monarch butterfly (*Danaus plexippus*), milkweed bugs, various clades of milkweed and dogbane beetles, the tiger keelback snake, and the African crested rat (Mohammadi et al. 2022). CGs are typically produced as cocktails of compounds with varying hydrophobicities and all inhibit the sodium pump (Na^+^/K^+^-ATPase), hindering its function and disrupting ion flow across membranes of cells in the nervous system and other tissues (Agrawal et al. 2012). Na^+^/K^+^-ATPases are highly conserved among animal species, making their interactions with CGs a powerful model system for studying processes central to coevolution and chemical ecology, including in the context of multitrophic interactions (Agrawal et al. 2012; Groen and Whiteman 2021).

Milkweeds are common in North and Central America and serve as an important food source for a variety of specialized herbivorous insect species. Previous studies have shown that several of these specialists, including the monarch butterfly, milkweed and dogbane beetles, and the large milkweed bug (*Oncopeltus fasciatus*), can sequester CGs in different compartments of their bodies for protection against natural enemies and that this is facilitated by TSI (Groen and Whiteman 2021; Agrawal et al. 2021, 2022; Zhen et al. 2012). TSI evolved in herbivores from at least six orders of insects and parallel TSI-conferring substitutions were found in the first extracellular loop of the Na^+^/K^+^-ATPase at sites 111, 119, and 122 (Table 1) (Karageorgi et al. 2019; Yang et al. 2019). While monarch caterpillars and beetle grubs contain high CG levels in their hemolymph, milkweed bugs form storage compartments in which, aided by transporters, they can store high concentrations of hydrophilic CGs (Bramer et al. 2015). Milkweed bugs further store hydrophobic CGs throughout the fat body (Duffey et al. 1978).

Like CG-sequestering insect herbivores, their predators and parasites must also confront high levels of the toxins. Nematodes of the genus *Steinernema* are entomopathogenic nematodes (EPNs) that parasitize insects. They can be found in soil as infective juveniles (IJs) or within their insect hosts where they undergo further development (Dillman and Sternberg 2012). Milkweed and dogbane beetle larvae are present in the soil as root feeders (Agrawal & Hastings 2023; Peterson et al. 2001). Furthermore, monarch caterpillars and milkweed bugs regularly visit the soil as well, for example when disturbed (Barrett & Chiang 1967; McGruddy et al. 2021). When herbivorous insects move in or on soil, their risk of becoming infected by EPNs increases markedly. The species *S. carpocapsae* is known to occur in soil around milkweed plants (Erwin et al. 2014) and to naturally infect insects that feed on either belowground or aboveground plant parts (Peters 1996). While this species is a generalist, its presence near milkweed plants prompts the question of whether it can overcome the toxicity of CGs ingested by milkweed-feeding herbivores. This is particularly poignant in light of our recent finding that *S. carpocapsae*, but not any of its known congeners, evolved a substitution in the first extracellular loop of its Na^+^/K^+^-ATPase (N122H) (Table 1). This substitution was shown to have a large effect on TSI to CGs in insects and evolved in many insect herbivores specialized on milkweeds, suggesting a possibility of parallel evolution across trophic levels (Groen and Whiteman 2021). Our research addresses the following questions: (1) Does *S. carpocapsae* infect CG-carrying insects with more success than other *Steinernema* species that lack TSI? *I.e*., does CG sequestration by herbivorous insects influence EPN infection? (2) Does *S. carpocapsae* prefer or naturally navigate towards milkweed roots or milkweed-feeding insects by using CGs or other chemicals as cues?

**Table 1:**
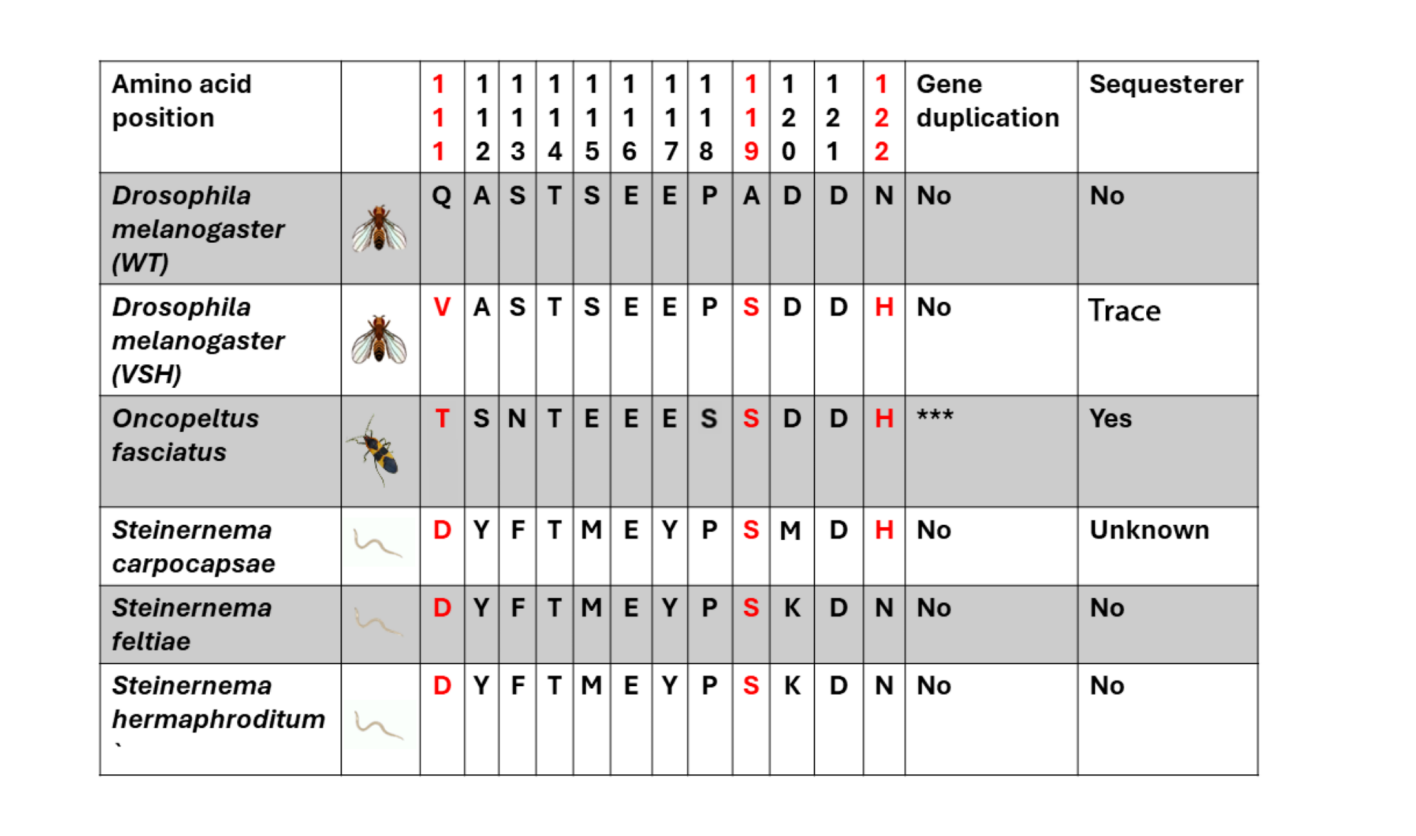
Amino acid sequences of the Na⁺/K⁺-ATPases of insects and nematodes studied here. The species include *Drosophila melanogaster* (wild type [WT] and mutant strain [VSH]), *Oncopeltus fasciatus*, *Steinernema carpocapsae*, *S. feltiae*, and *S. hermaphroditum*. The sequences at positions 111–122 of the Na⁺/K⁺-ATPase α subunit are shown (numbering relative to the pig ATP1A1 sequence). *Oncopeltus fasciatus* evolved four copies of the underlying gene, *ATPα1*, with the copy that evolved the largest number of substitutions and that is the most highly expressed across tissues (ATPα1B) shown here (Yang et al. 2019). Substitutions at positions 111, 119, and 122 in this region have been associated with TSI to CGs (shown in red), with substitution N122H having been associated with the ability to tolerate high levels of sequestered CGs (Agrawal et al. 2024; Karageorgi et al. 2019). EPN sequence data was acquired from the published genome sequences (Dillman et al. 2015)

## Materials and Methods

### EPN White Trap Set Up

For our experiments, we used nematodes of the following species in the genus *Steinernerma*: *S. bicornutum* (1298 strain), *S. carpocapsae* (All strain), *S. ceratophorum* (Chin strain), *S. diaprepesi* (Btw strain), *S. feltiae* (SN strain), *S. glaseri* (NC strain), *S. hermaphroditum* (CS34 strain), *S. khuongi* (Arc strain), and *S. riobrave* (355 strain). These EPNs have been kept in a laboratory environment for years to decades. We maintained nematode populations using an established white-trap protocol (McMullen and Stock 2014). Three or four wax worms (*Galleria mellonella*; Crittergrub) were placed onto p-8 filter paper in a 60-mm Petri dish. Then, 700-1,000 nematodes of a species were transferred onto the plate. Nematodes were given three days to infect the wax worms before being moved onto white traps. For this, 100-mm Petri dishes were used, each with a 30-mm Petri dish lid placed in the middle that was covered with p-5 filter paper. Wax worms were then moved onto the center of the paper and filtered tap water was used to fill enough of the larger Petri dish to get the filter paper wet along its sides. IJs were given five days to exit wax worms. Water was then collected to obtain healthy IJs and stored horizontally in cell culture flasks in a fridge at 16.7 °C. EPNs were stored for up to six months. Two weeks prior to the experiment, EPNs needed for the study were placed on fresh white traps.

### Large Milkweed Bug Maintenance and Culturing

*Oncopeltus fasciatus* milkweed bugs for the CG injection assay (see below) were ordered commercially (Carolina Biological Supply) and reared on sunflower seeds. They were maintained in an incubator at 26 °C with 65% humidity and a 16 h light / 8 h dark phase. Large containers with mesh lids were used as habitats for the bugs. A cotton ball was placed in each habitat for egg laying. Water was refilled continuously to ensure optimal conditions for the bugs. Bugs for the sunflower and milkweed seed assay were reared on sunflower seeds or seeds of the milkweeds *A. curassavica*, *A. incarnata*, *A. perennis*, and *A. syriaca*. Bug populations were established on seeds from these different plant species, after collecting wild *O. fasciatus* individuals from *A. syriaca* plants in Ithaca, NY (USA), and kept in the lab for fewer than eight generations.

### Large Milkweed Bug Injections

We used adult *O. fasciatus* bugs (Carolina Biological Supply), that were reared on sunflower seeds, for injections with saline, digoxin, or digitoxin to test if more hydrophobic CGs could influence infections with *S. carpocapsae* or *S. feltiae*. Although capable of infecting Drosophila (Huynh et al. 2023), S. *hermaphroditum* failed to infect milkweed bugs consistently, as did six other species of EPNs we tested: *S. riobrave*, *S. khuongi*, *S. glaseri*,* S. diaprepesi*, *S. ceratophorum*, and *S. bicornutum*. Bugs were collected and placed in a habitat with food, but no water, for 3–4 h prior to injections. Bugs were injected with saline solution with or without digoxin or digitoxin. Control saline solution was prepared by mixing 15% ethanol, 1% NaCl, and 10 mL of 0.3% DMSO dissolved in DI water. The toxin solutions were made by adding digoxin or digitoxin to this solution at a concentration of 2 mg mL^− 1^. Insects were immobilized by placing them at -20 °C for 2 min. Using a fine glass pipette, 3 µL of saline solution with or without toxin was injected into the milkweed bug haemocoel between sterna 5 and 6, making sure to face away from the head. Bugs were then placed back under light with food and water until they had recovered. Bugs were considered to have recovered when they were able to walk around the habitat (Duffey et al. 1978; Lohr et al. 2017). Bugs were then used for EPN infection assays (see below).

### Nematode Infections of Large Milkweed Bugs

Milkweed bugs fed different diets or injected with different CGs were infected with EPNs using the white trap set ups described above, with the bugs replacing the wax worms. Three bugs were placed per white trap with filter paper. Bugs were infected with *S. carpocapsae* or *S. feltiae. Steinernema khuongi*, *S. diaprepesi*, *S. ceratophorum*, *S. bicornutum*, *S. riborave*, *S. glaseri*, and *S. hermaphroditum* failed at infecting bugs consistently and were not used for experiments any further. Nematodes were collected and suspensions of 2 IJs per µL were prepared. Each plate was infected with 250 µL of nematode suspension and monitored over subsequent days. The number of days until milkweed bug death were counted until all bugs had died or until bug dissections were performed at nine days post infections. Milkweed bugs were dissected in water and data was recorded for the number of bugs that were killed, the presence of nematodes, the number of nematode progeny, the growth of the nematodes’ bacterial symbionts, the presence of lipid droplets, the distribution of nematodes across life stages, and the number of nematodes that exhibited a coiling phenotype, which is an indication of neurotoxicity (Harrington et al. 2023; Supplementary Fig. 1, Supplementary Fig. 2).

### Drosophila Maintenance and Culturing

A mutant strain of *Drosophila melanogaster* (vinegar fly, Drosophila), w[*]; Atpalpha[VSH], with amino acid substitutions in ATPα in the w[1118] background was obtained from the Bloomington Drosophila Stock Center. Using CRISPR genome engineering, these flies were previously endowed with three Na^+^/K^+^-ATPase substitutions that evolved in the monarch butterfly (Q111V, A119S, and N122H) and that confer TSI to CGs (Karageorgi et al. 2019). Flies were maintained in vials containing regular fly food (Glucose Food; Archon Scientific) in an incubator at 26 °C with a 12 h light / 12 h dark phase. Flies were sorted with 22 adult males and 22 adult females into new fly food vials every other week to ensure optimal growth conditions.

### Nematode Infections of Drosophila Larvae on Ouabain-Containing Diet

Second-instar larvae of the Drosophila mutant strain w[*]; Atpalpha[VSH] were placed on filter paper or agar and subsequently exposed to IJs of *S. carpocapsae*,* S. feltiae* or S. *hermaphroditum* (Supplementary Fig. 3). Drosophila was previously shown to be susceptible to all three of these EPN species (Huynh et al. 2023; Dobes et al. 2012; Peña et al. 2015). Larvae were fed a non-toxic diet or a diet containing the purified CG ouabain (Sigma-Aldrich). We chose the hydrophilic CG ouabain since we could deliver millimolar levels of this CG into this non-CG-sequestering insect via its diet to mimic the high CG levels that can be found in the hemolymph of monarch caterpillars without needing levels of a solvent such as DMSO that would have adverse effects on insects and nematodes (AlOkda and Van Raamsdonk 2022; Groen et al. 2017; Karageorgi et al. 2019). Fly food containing ouabain was created by preparing Nutri-Fly food packets (Genesee) in a flask. Once cooled, 15 mM of ouabain was added to the flask. Fly food was poured into vials and allowed time to cool, then stored at 4 °C. Non-toxic fly food, prepared as described above without the addition of ouabain, served as the control. Six to eight adult males and six to eight adult females were placed into each vial and allowed to mate for 3–4 days. Adults were removed and larvae were left to hatch. Twelve-well plates were prepared with filter paper or NGM agar and one second-instar fly larva in each well. Twenty EPNs (*S. carpocapsae*, *S. feltiae* or *S. hermaphroditum*) in 10 µL of water were then added to the wells with fly larvae that were either feeding on non-toxic or ouabain-containing food. Plates were covered with parafilm. Larval mortality was recorded at 2, 12, 24, and 48 h post infection to assess whether CGs influence the ability of EPNs to kill their insect hosts. The experiment was performed in triplicate.

### Milkweed Root Extract Collections

*Asclepias curassavica* seeds were germinated in seedling trays containing organic planting mix (Sun-Gro Horticulture). Seedlings were maintained in a growth chamber at 26 °C with a 16 h light / 8 h dark phase at a light intensity of 200 µM m^–2^ s^–1^ until the first true leaf was observed. Seedling roots were then thoroughly rinsed with water to eliminate soil particles. Subsequently, the roots were flash-frozen in liquid nitrogen and pulverized into a fine powder using a mortar and pestle. The resulting powdered root tissues were then dissolved in three volumes of 5% methanol (MeOH) solution and centrifuged at 10,000 g for 10 min. The resulting supernatant was carefully removed, and the pellet was suspended in H_2_O before being stored at 4 °C until further use (Liu et al. 2019).

### Chemotaxis Assays with Root Extracts on Plates

Chemotaxis plates were set up according to a previously published protocol (Liu et al. 2019). Chemotaxis agar media was poured into small Petri dishes (50-mm diameter). Then, using a pipette, a small crater-like shape was created in the middle, forming a higher level of agar referred to as the volcano deck. IJs were exposed to wax worm host cuticle for a duration of 20 min to allow host stimulation, which allows for higher participation rates of EPNs (Baiocchi et al. 2019). A quantity of 20 µL of root extract was placed onto the volcano deck, followed by the addition of 4 µL of the paralytic agent sodium azide (NaN_3_) at 0.5 M. The NaN_3_ solution was made by adding 500 µL of 1-M stock to 500 µL of milliQ water. The paralytic agent was used to visualize the EPNs’ initial directional movement. Subsequently, a suspension containing 100–200 IJs in 20 µL of H_2_O was carefully dispensed around the perimeter of the deck slope. Plates were then stacked into groups of three in opposite orientations, placed in a box with a lid on a vibration-resistant platform and stored in the dark for 24 h. After 24 h, the numbers of IJs on and below the deck as well as the number of IJs that displayed a coiling phenotype were recorded. The experiment was performed in triplicate.

### Preference Chemotaxis Assay for Milkweed Roots in Sand

Sand was autoclaved and then washed repeatedly with tap water followed by DI water. The assays were performed in olfactometers (Supplementary Fig. 4a), the setup of which has been described previously (Wu and Duncan 2020). The measurements of the pipes and glass were as follows: each tube measured 9 cm in diameter and 5 cm in length, the pipe was 7 cm in length and 6 cm in height, and the entire set-up was 15 cm in length. Sand was dried at 60 °C overnight and moistened to 12% with tap water. Filter paper was spotted with 20 µL of tap water as a control or with 20 µL of test solution, and then placed at the end of the tubes. A consistent weight of 28 g of sand per tube was used for each replicate and trial. Root extracts were protected from light. Prenol is a known repellent for EPNs and served as a negative control test solution (Baiocchi et al. 2017). A 2-M solution of prenol was made by adding 203 µL of prenol (99.9% purity) to 797 µL of DI water. *Asclepias curassavica* milkweed root extracts were prepared using the method described above. The region near the middle of the olfactometer consisted of sand moistened to 12% with tap water, which was the same as the conditions on the control side of the olfactometer to ensure that no biases were introduced. One thousand IJs in 100 µL of H_2_O were carefully dispensed into the center of the olfactometer. IJs were collected from fresh white traps followed by host stimulation prior to each experiment. For host stimulation, three wax worms were placed on Petri dishes with filter paper and the IJs were allowed to interact with host cuticle for 15–20 min before being collected and used (Baiocchi et al. 2019). Each olfactometer was placed horizontally on a foam pad to suppress any vibrations. These were then placed in the dark in random orientations to avoid any potential directionality biases. Each assay ran for 24 h before the caps were removed from each side of the olfactometer separately. Nematodes were collected using the Baermann funnel technique followed by counting their numbers. Each replicate had three biological replicates for each condition and each EPN species. Choice percentages were calculated by counting the number of nematodes in the control area or the test area, dividing these by the total number of IJs inoculated, and multiplying the result by 100. The experiment was performed in triplicate within each of three biological replicates. The figures were graphed using means across biological replicates, and a chi-squared analysis was conducted on the average of each replicate, with the number in the control treatment serving as the expected value and the number in the test treatment serving as the observed value.

### Chemotaxis Assays with Purified Chemicals

Chemotaxis plates were prepared as described previously (Dillman et al. 2012; Baiocchi et al. 2017): 17 g agar was dissolved in 1,000 mL dH_2_O and autoclaved for 30 min; this was followed by the addition of 5 mL filtered potassium phosphate buffer, 1 mL filtered MgSO₄, and 1 mL filtered CaCl₂. Plates were left at room temperature for 12 h before the experiment. EPNs were collected from fresh white traps (within two weeks old) to ensure healthy IJs were used in assays. IJs were collected and washed with DI water before 500 µL of IJ suspension at a density of one IJ per µL was placed onto a wax worm. IJs were given 20 min to have contact with the host cuticle for host stimulation (Baiocchi et al. 2019). They were then collected and left at a density of eight IJs per µL, ready to be used for chemotaxis assays. A 2-M solution of the EPN repellent prenol was made by adding 203 µL of prenol (99.9% purity) to 797 µL of DI water. A tetrahydrofuran (THF) solution was made by adding 14.4 µL of THF to 985.6 µL of DI water. THF is a known attractant for *S. carpocapsae* and *S. feltiae* (Dillman et al. 2012). A 0.5-M solution of the paralytic NaN_3_ was made by adding 500 µL of 1-M stock to 500 µL of milliQ water. Ouabain solutions of 15 mM or 100 µM were made by dissolving ouabain in DI water with 0.3% DMSO. Templates for chemotaxis assays were printed and placed under each chemotaxis plate. On the test side, 5 µL of chemical solution was placed in the test circle. On the control side, 5 µL of DI water with 0.3% DMSO was placed in the control circle. Chemicals were given 15–20 min to diffuse. Then, 2 µL of 0.5 M NaN_3_ was placed in each scoring circle. A 15-µL suspension of IJs at a density of 5 IJs per µL was placed in the center of the plate, containing a total of 70–170 nematodes. Plates were then stacked into groups of three in opposite orientations, placed in a box with a lid on a vibration-resistant platform and stored in the dark. The assay was run for two hours, after which data was taken on where nematodes were found: the test side, the control side or in the middle. Choice percentages were calculated by counting the number of nematodes in the control area or the test area, dividing these by the total number of IJs inoculated, and multiplying the result by 100. The experiment was performed in triplicate within each of three biological replicates. The figures were graphed using means across biological replicates, and a chi-squared analysis was conducted on the average of each replicate, with the number in the control area serving as the expected value and the number in the test area serving as the observed value.

### Graphing and Statistical Analysis

All graphs were made using PRISM with the mean and standard error of the mean (SEM) of each treatment group. Statistical analysis was performed using one-way or two-way ANOVA followed by a post-hoc Tukey’s analysis or using chi-squared analysis depending on the dataset. Figures were assembled using Adobe Photoshop.

## Results

### *Steinernema carpocapsae* Infections Show Little Impediment by CGs in Milkweed Bugs

**Fig. 1:**
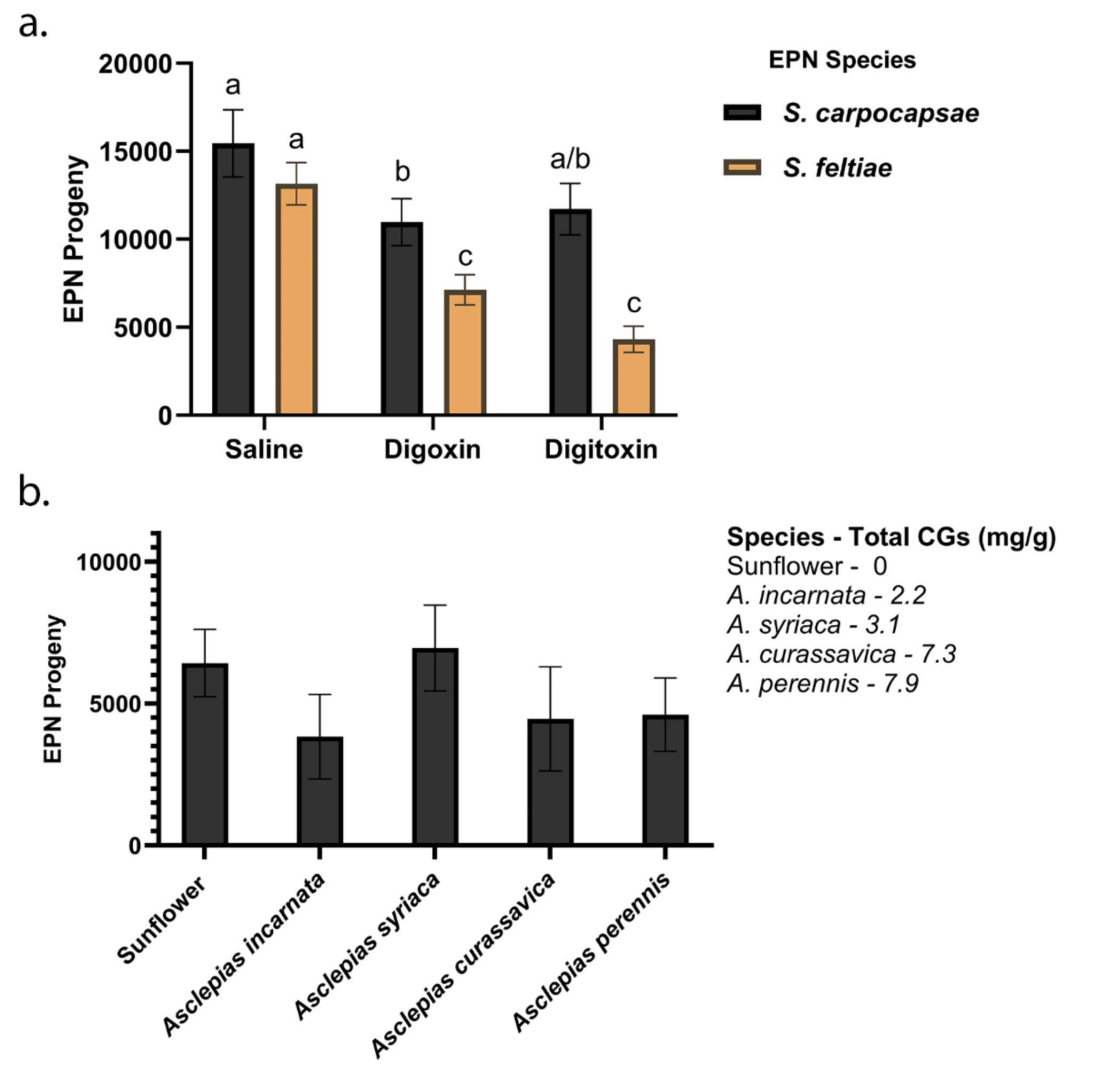
*Steinernema carpocapsae* has a significantly higher infection rate in CG-resistant large milkweed bugs injected with CGs than *S. feltiae*. **a.***S. carpocapsae* and *S. feltiae* progeny numbers at nine days post infection in dissected adult milkweed bugs injected with saline, digoxin or digitoxin. **b.***Steinernema carpocapsae* progeny numbers at nine days post infection in dissected adult milkweed bugs reared on various seed diets. Statistical analysis was performed using two-way ANOVA **(a)** and one-way ANOVA **(b)** followed by post-hoc Tukey’s analysis. Data are represented as mean ± SEM

*Steinernema feltiae* could infect, kill, and reproduce inside milkweed bugs, but showed a significant decrease (46% and 67% respectively) in fecundity inside bugs injected with digoxin (*p* < 0.001) or digitoxin (*p* < 0.001; Fig. 1a). Digoxin and digitoxin also caused significant increases in the numbers of nematodes showing a coiling phenotype, which is a sign of neurotoxicity (Supplementary Fig. 1, Supplementary Fig. 2). *Steinernema carpocapsae* showed no decrease in progeny numbers when infecting insects injected with digitoxin (*p* > 0.05) but showed a slight (29%) decrease with digoxin (*p* = 0.042). However, *S. carpocapsae* had more progeny than *S. feltiae* when bugs had been injected with either digoxin (*p* = 0.039) or digitoxin (*p* < 0.001), despite showing a 153% increase in the number of nematodes showing a coiling phenotype in the presence of digoxin (Supplementary Fig. 1, Supplementary Fig. 2).

### *Steinernema carpocapsae* Infects Milkweed Bugs Reared on a Variety of Milkweed Species

The fecundity of *S. carpocapsae* showed little impediment by injections of adult milkweed bugs with purified CGs and next we tested if *S. carpocapsae* infections of *O. fasciatus* were influenced by bug sequestration of mixtures of CGs from seeds of a variety of milkweed species (Fig. 1b). No significant difference was found in *S. carpocapsae* fecundity when infecting milkweed bugs reared on various seed diets (*p* > 0.05). However, the nematodes did show a 40% increase in coiling phenotypes when bugs were reared on seeds of *A. perennis*, a milkweed species that is known to accumulate a relatively high level and diversity of CGs (Rasmann and Agrawal 2011; Supplementary Fig. 1c, Supplementary Fig. 2).

### *Steinernema carpocapsae* Causes Higher Mortality than Other EPNs in CG-Containing Drosophila Hosts

**Fig. 2:**
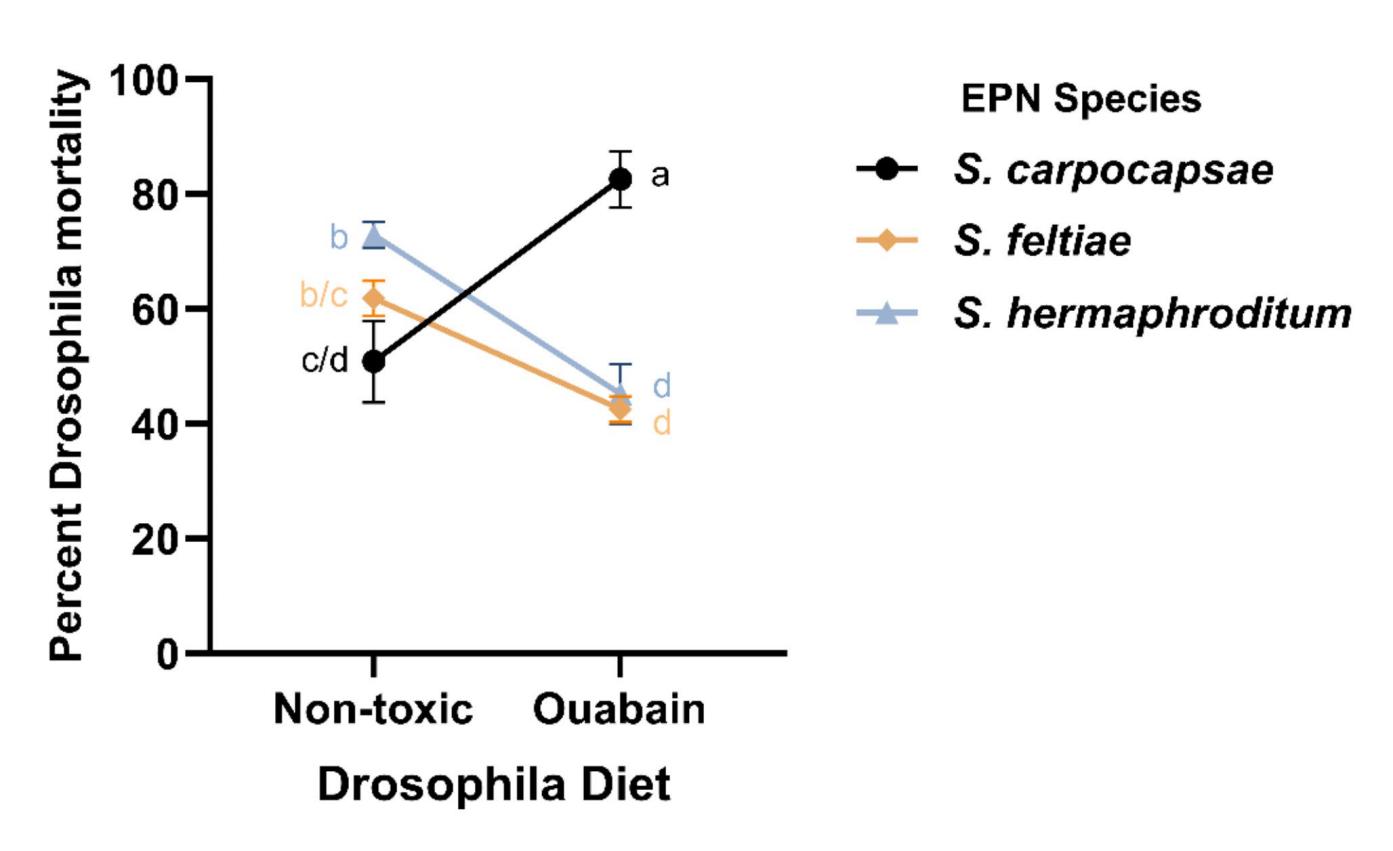
*Steinernema carpocapsae* causes higher mortality of CG-insensitive Drosophila mutants when these are reared on a diet containing ouabain. Percent mortality of mutant Drosophila larvae reared on a non-toxic or ouabain-containing media at 48 h post infection with *S. carpocapsae*,* S. feltiae*, and *S. hermaphroditum* on filter paper. Data are represented as mean ± SEM. Statistical analysis was performed using two-way ANOVA followed by post-hoc Tukey’s tests

Infections on filter paper resulted in 63% higher nematode infectivity and insect host mortality than infections on agar across all EPN species (Supplementary Fig. 3a). After having established this, we then observed if EPN virulence was impacted by whether Drosophila larvae were fed a non-toxic or an ouabain-containing diet. *Steinernema carpocapsae* caused 63% higher mortality when ouabain was present in its host’s diet on filter paper (*p* < 0.001) and on agar (*p* = 0.016; Fig. 2, Supplementary Fig. 3c). Conversely, *S. feltiae* and *S. hermaphroditum* both caused less mortality when ouabain had been added to their hosts’ diets on filter paper (*p* = 0.011 and *p* < 0.001, respectively), but showed no difference on agar (Fig. 2, Supplementary Fig. 3c).

### *Steinernema carpocapsae* is Attracted to Milkweed Root Extract on Agar Plates

**Fig. 3:**
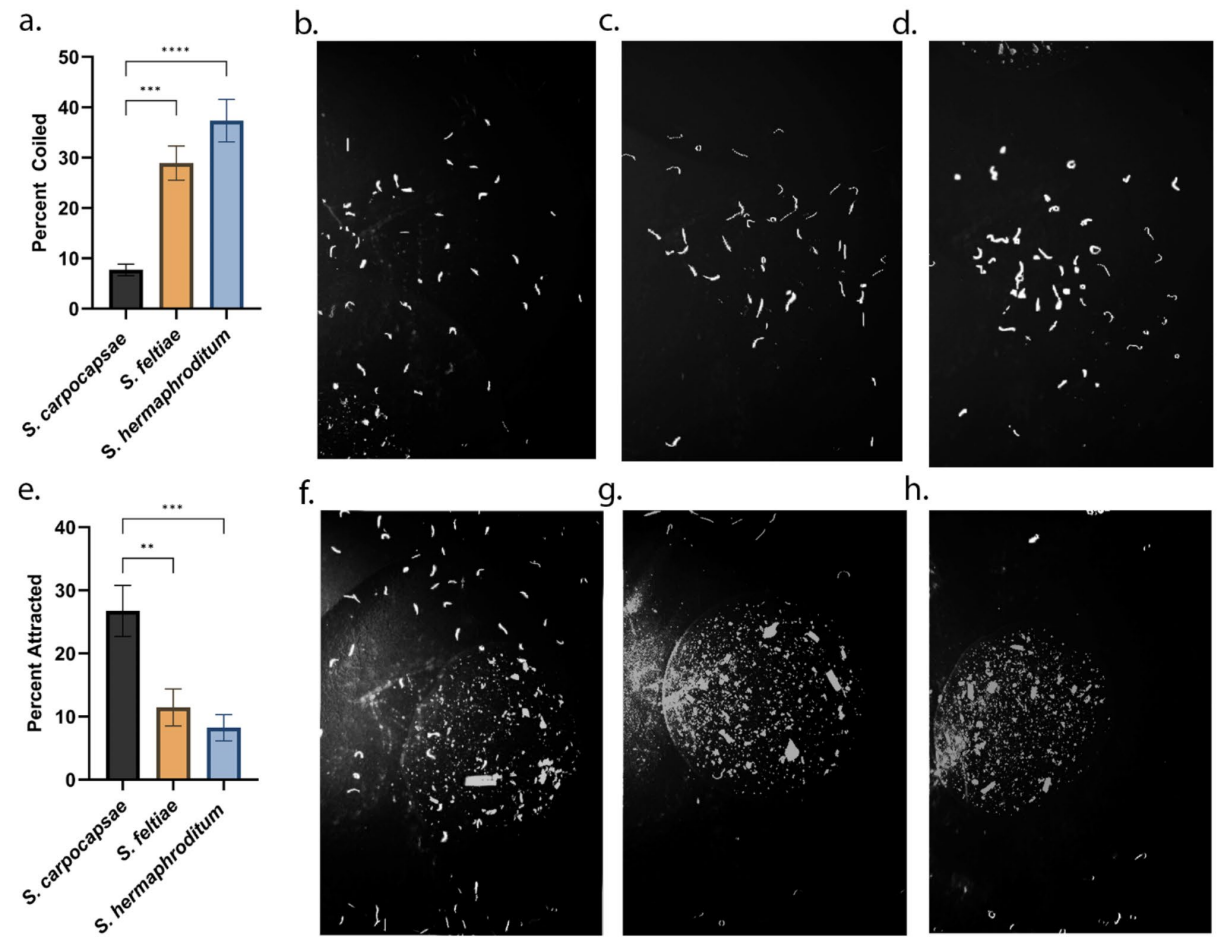
*Steinernema carpocapsae* is unaffected by and possibly attracted to *Asclepias curassavica* root extract. **a.** Percent of EPNs demonstrating a coiling phenotype (a c-shaped body posture) when near *A. curassavica* root extract. **b-d.***S. carpocapsae*, *S. feltiae*, and *S. hermaphroditum* (consecutively) below and around decks with *A. curassavica* root extract and demonstrating a coiling phenotype. **e.** Percent of EPNs found on decks of agar plates containing *A. curassavica* root extract. **f-h.***Steinernema carpocapsae*, *S. feltiae*, and *S. hermaphroditum* (consecutively) on decks with root extract. Data are represented as mean ± SEM. Statistical analysis was performed using one-way ANOVA followed by post-hoc Tukey’s analysis. Images were adjusted with Adobe Photoshop

We then tested if variation in sensitivity to CGs among the EPN species is associated with variation in attraction towards CGs. A behavioral assay was conducted on agar plates to determine attraction of *S. carpocapsae*,* S. feltiae*, and *S. hermaphroditum* to *A. curassavica* root extract. *Steinernema feltiae and S. hermaphroditum* displayed a coiling phenotype significantly more often than *S. carpocapsae* (*p* < 0.001 and *p* < 0.001, respectively), which is indicative of stronger neurotoxic effects of CGs on the first two species (Fig. 3a-d). In addition, *S. carpocapsae* exhibited significantly stronger attraction towards the root extract than *S. feltiae* and *S. hermaphroditum* (*p* = 0.004 and *p* < 0.001, respectively; Fig. 3e-h).

### *Steinernema carpocapsae* is Attracted to Milkweed Root Extract in Sand

**Fig. 4:**
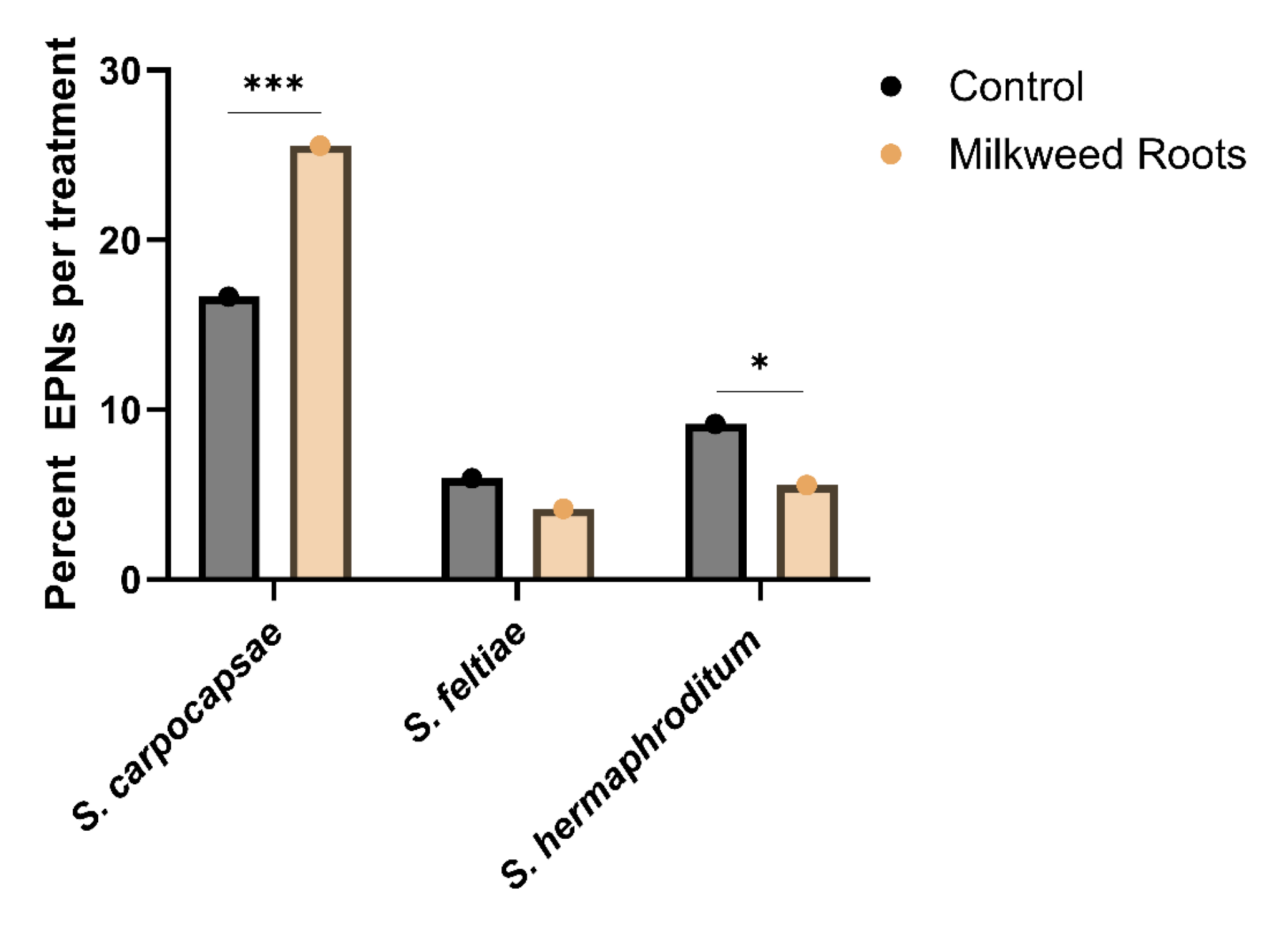
*Steinernema carpocapsae* shows attraction to milkweed root extract in sand. Choice percentages of *S. carpocapsae*,* S. feltiae*, and *S. hermaphroditum* for *Asclepias curassavica* root extract or control solution are shown. Data are represented as mean percentages of three replicate trials with three technical replicates per trial. Statistical analysis was performed using chi-squared tests on choice values from each individual trial with the control treatment providing the expected values and the test treatments providing the observed values

We next tested attraction to milkweed root extract in a behavioral assay in sand (Supplementary Fig. 4), which is more representative of soil environments than agar. *Steinernema carpocapsae* again showed substantial attraction to *A. curassavica* root extract (*p* < 0.0001), whereas *S. feltiae* was indifferent (*p* = 0.438) and *S. hermaphroditum* was repelled (*p* = 0.026; Fig. 4). EPNs behaved as expected in both the control (tap water-tap water) and the negative control (tap water-prenol, a known EPN repellent) experiments (Dillman et al. 2012; Baiocchi et al. 2017), demonstrating no preference in the control (*p* ≥ 0.494; Supplementary Fig. 4a) and strong repulsion in the negative control (*p* < 0.001; Supplementary Fig. 4b).

### *Steinernema carpocapsae* is Attracted to Ouabain

**Fig. 5:**
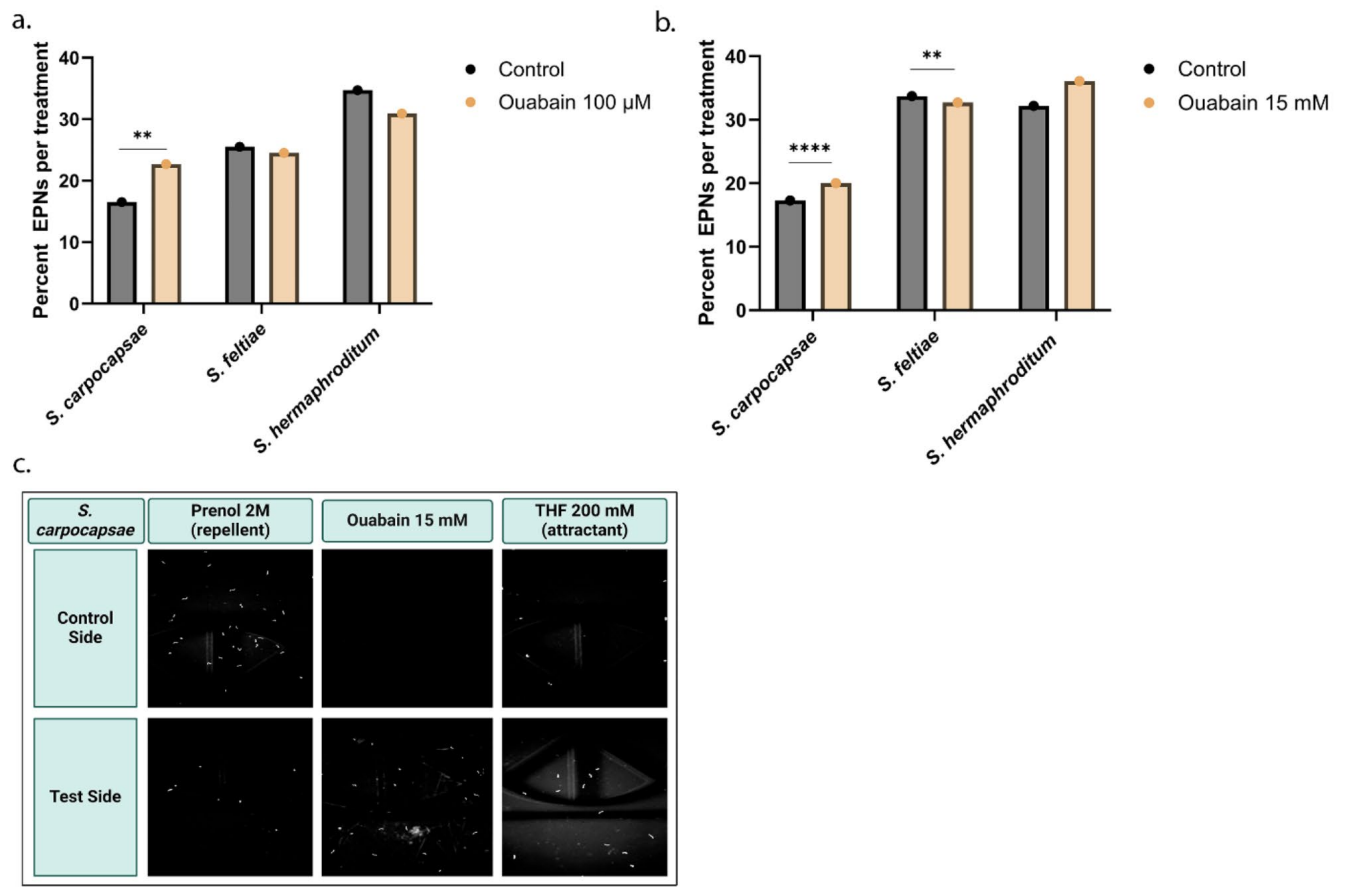
*Steinernema carpocapsae* is attracted to ouabain in an assay on agar. **(a)** Choice percentages of *S. carpocapsae*,* S. feltiae*, and *S. hermaphroditum* with ouabain at 100 µM. **(b)** Choice percentages of *S. carpocapsae*,* S. feltiae*, and *S. hermaphroditum* with ouabain at 15 mM. **(c)** Images of *S. carpocapsae* on the control side or test side of chemotaxis assays against the odorants prenol (negative control) and THF (positive control) as well as ouabain at 15 mM. Data are represented as mean percentages of three replicate trials with three technical replicates per trial. Statistical analysis was performed using chi-squared tests on choice values from each individual trial with the control treatment providing the expected values and the test treatments providing the observed values. Images were adjusted with Adobe Photoshop

Given the attraction of *S. carpocapsae* towards milkweed root extract, we aimed to see if this attraction may be caused by CGs. The behavioral responses of *S. carpocapsae*,* S. feltiae*, and *S. hermaphroditum* were assessed with different compounds (Fig. 5 and Supplementary Fig. 5), including the CG ouabain, prenol as a repellent, and the known EPN attractant THF (Dillman et al. 2012; Baiocchi et al. 2017). All EPNs showed repulsion to prenol as expected (*p* < 0.001). *Steinernema carpocapsae* and *S. feltiae* showed mild attraction to THF (*p* = 0.003 for *S. carpocapsae* and *p* < 0.001 for *S. feltiae*), while *S. hermaphroditum* had no strong response (*p* = 0.022). *Steinernema carpocapsae* was the only species that displayed significant attraction to ouabain at 100 µM (*p* = 0.003 for *S. carpocapsae*, *p* = 0.271 for *S. feltiae*, and *p* = 0.867 for *S. hermaphroditum*; Fig. 5a). In addition, *S. carpocapsae* showed strong attraction towards ouabain at 15 mM (*p* < 0.001), with *S. feltiae* showing mild repellence (*p* = 0.008) and *S. hermaphroditum* showing no differential response (*p* = 0.271; Fig. 5b, c).

## Discussion

Through a series of infection and behavioral assays we observed significant differences in the patterns of sensitivity and attraction of *Steinernema* EPN species towards toxic CGs that are present in and around milkweeds and the specialist insect herbivores that sequester them. We developed two novel EPN infection assays to investigate the influence of CGs on the infection process.

The first assay made use of a hemimetabolous insect that is also a natural specialist herbivore of milkweeds, the CG-sequestering large milkweed bug *O. fasciatus*. The large milkweed bug has long been studied as a model system to elucidate mechanisms underlying the development of hemimetabolous insects as well as CG sequestration. Following established protocols (Duffey et al. 1978; Lohr et al. 2017), milkweed bugs were reared on sunflower seeds before receiving injections with saline, digoxin or digitoxin upon reaching adulthood. *Steinernema feltiae* showed a significant reduction in fecundity when infecting bugs that were injected with digoxin and an even more significant reduction with digitoxin. *Steinernema carpocapsae* showed no significant reduction in fecundity when confronted with digitoxin but showed a slight decrease in fecundity with digoxin. The negative effect of digoxin on *S. carpocapsae* also became apparent by an increase in frequency of worms displaying the coiling phenotype, indicating that some CGs could have minor toxic effects on this EPN.

We then reared milkweed bugs on seeds of a diverse set of milkweed species that contained different levels and mixtures of CGs with varying hydrophobicities and infected these bugs with *S. carpocapsae*. The nematodes showed no significant differences in fecundity across the insects reared on different diets and only showed an increased frequency in the occurrence of the coiling phenotype in bugs reared on seeds of *A. perennis*. Interestingly, this species is known to accumulate a relatively high level and diversity of CGs among milkweed species that have been profiled (Rasmann and Agrawal 2011). Previous studies have shown that CGs in the seeds can even have a negative impact on specialized insects like the large milkweed bug (Agrawal et al. 2022). Our data suggest that seed CGs sequestered by *O. fasciatus* may have minor negative effects on the EPN *S. carpocapsae* and much more pronounced negative effects on its congener *S. feltiae*.

To further investigate the effects of more hydrophilic CGs on insect infections by different species of *Steinernema*, we developed a second infection assay. We modeled infections of holometabolous insects such as coleopterans, dipterans, and lepidopterans that feed on milkweeds by using gene-edited Drosophila larvae with TSI substitutions in their Na^+^/K^+^-ATPase. The TSI substitutions dramatically increase the CG resistance of these flies, and the use of their larvae allowed EPN infections to take place in the presence of millimolar levels of hydrophilic CGs, which is similar to levels of such CGs observed in the hemolymph of monarch butterfly caterpillars (Agrawal et al. 2012). The presence of ouabain in Drosophila larvae did not negatively impact infections by *S. carpocapsae*, but did affect the virulence of *S. feltiae* and *S. hermaphroditum*. This suggested that *S. carpocapsae* was less sensitive to the toxic effects of CGs than the other two EPN species. Similar differences between EPN strains were previously observed for *Heterorhabditis* spp. infections of the western corn rootworm, where strains varied in their levels of behavioral and metabolic resistance to the toxic benzoxazinoids the insect sequesters from maize (Zhang et al. 2019). In our experiments, *S. carpocapsae* even increased the mortality of Drosophila larvae when they were fed an ouabain-containing diet relative to a control diet, suggesting that *S. carpocapsae* might be attracted to CGs.

*Steinernema carpocapsae* has been observed around milkweed plants (Erwin et al. 2014) and evolved substitutions in its Na^+^/K^+^-ATPase similar or identical to those found in many specialized milkweed-feeding insects (Groen and Whiteman 2021). This nematode showed attraction to milkweed root extract on agar plates and in sand as well as to the purified CG ouabain on agar plates. *S. carpocapsae* attraction to CGs was apparent at both the millimolar levels of CGs that may be found in sequestering herbivorous insect prey and the micromolar levels of CGs that may be found in the insects’ milkweed host plants. This behavior may therefore provide insight into why *S. carpocapsae* has been found near milkweed plants: CGs might help it navigate toward the plant’s chemical cues in root secretions or toward insects that sequester the toxins, such as monarch caterpillars, milkweed bugs or grubs of milkweed or dogbane beetles. Such CG-directed navigation toward prey could give *S. carpocapsae* a competitive advantage over other EPNs in the vicinity of milkweed plants. However, more research will be necessary in these and other interactions to establish if the preferences that different EPN species exhibit for certain plant species may correlate with the presence of their preferred insect herbivore hosts (Zhang et al. 2021).

Our results suggest that *S. carpocapsae* is positively affected by the presence of CGs when infecting insects that sequester them, unlike species such as *S. feltiae* and *S. hermaphroditum*, which faced varying degrees of neurotoxicity and fitness loss depending on the CGs encountered and the prey being infected. Our observations suggest that *S. carpocapsae* may have evolved mechanisms—specifically, target site insensitivity through mutations in the Na⁺/K⁺ ATPase—that confer insensitivity to the toxic effects CGs have on many animals. This toxin resistance could potentially benefit *S. carpocapsae* by enabling it to target insects on which it has higher fecundity compared to other EPN species. Investigating the mechanisms and evolution of resistance to CGs in *S. carpocapsae* will be a worthwhile endeavor.

Multitrophic interactions extend beyond the realm of the aboveground domain, and there is much left to be done to integrate the belowground domain into our picture of such interactions (Zhang et al. 2021). When subjected to arthropod herbivore attacks, plants frequently change emission profiles of volatile and non-volatile compounds, which can attract natural enemies of these herbivores, including EPNs (Ali et al. 2011; Ceballos et al. 2023; Degenhardt et al. 2009; Rasmann et al. 2005). Our findings indicate an attraction of the EPN *S. carpocapsae* to both the purified CG ouabain and milkweed root extract, warranting further investigation.

## Electronic Supplementary Material

Below is the link to the electronic supplementary material.

Supplementary Material 1

Supplementary Material 2

## Data Availability

Data is provided within the manuscript or supplementary information files.

## References

[CR3] Agrawal AA, Hastings AP (2023) Tissue-specific plant toxins and adaptation in a specialist root herbivore. Proc Natl Acad Sci U S A 120:e2302251120 37216531 10.1073/pnas.2302251120 PMC10235950

[CR5] Agrawal AA, Petschenka G, Bingham RA, Weber MG, Rasmann S (2012) Toxic cardenolides: chemical ecology and coevolution of specialized plant-herbivore interactions. New Phytol 194:28–45 22292897 10.1111/j.1469-8137.2011.04049.x

[CR1] Agrawal AA, Boroczky K, Haribal M, Hastings AP, White RA, Jiang RW, Duplais C (2021) Cardenolides, toxicity, and the costs of sequestration in the coevolutionary interaction between monarchs and milkweeds. Proc Natl Acad Sci U S A 118:e2024463118 33850021 10.1073/pnas.2024463118 PMC8072370

[CR2] Agrawal AA, Espinosa L, Del Alba X, Lopez-Goldar AP, Hastings RA, White R, Halitschke S, Dobler G, Petschenka, Duplais C (2022) Functional evidence supports adaptive plant chemical defense along a geographical cline. Proc Natl Acad Sci U S A 119:e2205073119 35696564 10.1073/pnas.2205073119 PMC9231628

[CR4] Agrawal AA, Hastings AP, Lenhart PA, Blecher M, Duplais C, Petschenka G, Hawlena D, Wagschal V, Dobler S (2024) Convergence and divergence among Herbivorous insects Specialized on toxic plants: revealing syndromes among the Cardenolide feeders across the Insect Tree of Life. Am Nat 204:201–220 39179235 10.1086/731277

[CR6] Ali JG, Alborn HT, Stelinski LL (2011) Constitutive and induced subterranean plant volatiles attract both entomopathogenic and plant parasitic nematodes. J Ecol 99:26–35

[CR7] AlOkda A, Van Raamsdonk JM (2022) Effect of DMSO on lifespan and physiology in C. Elegans: implications for use of DMSO as a solvent for compound delivery. MicroPubl Biol 2022:1017912micropubbiology000634 10.17912/micropub.biology.000634 PMC9494168 36158529

[CR9] Baiocchi T, Lee G, Choe DH, Dillman AR (2017) Host seeking parasitic nematodes use specific odors to assess host resources. Sci Rep 7:6270 28740104 10.1038/s41598-017-06620-2 PMC5524962

[CR8] Baiocchi T, Braun L, Dillman AR (2019) Touch-stimulation increases host-seeking behavior in Steinernema Carpocapsae. J Nematol 51:1–5 31814369 10.21307/jofnem-2019-067 PMC6909391

[CR10] Barrett RW, Chiang HC (1967) Changes of Behavior Pattern within the Fifth Nymphal Instar of the milkweed bug, Oncopeltus fasciatus (Dallas). Am Midl Nat 78:359–368

[CR11] Bramer C, Dobler S, Deckert J, Stemmer M, Petschenka G (2015) Na+/K+-ATPase resistance and cardenolide sequestration: basal adaptations to host plant toxins in the milkweed bugs (Hemiptera: Lygaeidae: Lygaeinae). Proc Biol Sci 282:20142346 25808891 10.1098/rspb.2014.2346 PMC4389604

[CR12] Ceballos R, Palma-Millanao R, Navarro PD, Urzua J, Alveal J (2023) Positive chemotaxis of the entomopathogenic nematode Steinernema Australe (Panagrolaimorpha: Steinenematidae) towards high-bush blueberry (Vaccinium corymbosum) root volatiles. Int J Mol Sci 24:10536 37445712 10.3390/ijms241310536 PMC10341914

[CR13] Degenhardt J, Hiltpold I, Kollner TG, Frey M, Gierl A, Gershenzon J, Hibbard BE, Ellersieck MR, Turlings TC (2009) Restoring a maize root signal that attracts insect-killing nematodes to control a major pest. Proc Natl Acad Sci U S A 106:13213–13218 19666594 10.1073/pnas.0906365106 PMC2726344

[CR16] Dillman AR, Sternberg PW (2012) Entomopathogenic nematodes. Curr Biol 22:R430–R431 22677279 10.1016/j.cub.2012.03.047 PMC4662870

[CR14] Dillman AR, Guillermin ML, Lee JH, Kim B, Sternberg PW, Hallem EA (2012) Olfaction shapes host–parasite interactions in parasitic nematodes. Proc Natl Acad Sci U S A 109:E2324–E2333 22851767 10.1073/pnas.1211436109 PMC3435218

[CR15] Dillman AR, Macchietto M, Porter CF, Rogers A, Williams B, Antoshechkin I, Lee M-M, Goodwin Z, Lu X, Lewis EE, Goodrich-Blair H, Stock SP, Adams BJ, Sternberg PW, Mortazavi A (2015) Comparative genomics of Steinernema reveals deeply conserved gene regulatory networks. Genome Biol 16:200 26392177 10.1186/s13059-015-0746-6 PMC4578762

[CR17] Dobes P, Wang Z, Markus R, Theopold U, Hyrsl P (2012) An improved method for nematode infection assays in Drosophila larvae. Fly 6:75–79 22614785 10.4161/fly.19553 PMC3397922

[CR18] Duffey SS, Blum MS, Isman MB, Scudder GGE (1978) Cardiac glycosides: a physical system for their sequestration by the milkweed bug. J Insect Phys 24:639–645

[CR19] Erwin AC, Züst T, Ali JG, Agrawal AA (2014) Above-ground herbivory by red milkweed beetles facilitates above‐ and below‐ground conspecific insects and reduces fruit production in common milkweed. J Ecol 102:1038–1047

[CR20] Gols R (2014) Direct and indirect chemical defences against insects in a multitrophic framework. Plant Cell Environ 37:1741–1752 24588731 10.1111/pce.12318

[CR22] Groen SC, Whiteman NK (2021) Convergent evolution of cardiac-glycoside resistance in predators and parasites of milkweed herbivores. Curr Biol 31:R1465–R1466 34813747 10.1016/j.cub.2021.10.025 PMC8892682

[CR23] Groen SC, Whiteman NK (2022) Ecology and evolution of secondary compound detoxification systems in caterpillars. In: Marquis RJ, Koptur S (eds) Caterpillars in the middle: tritrophic interactions in a changing world. Springer International Publishing, pp 115–163

[CR21] Groen SC, LaPlante ER, Alexandre NM, Agrawal AA, Dobler S, Whiteman NK (2017) Multidrug transporters and organic anion transporting polypeptides protect insects against the toxic effects of cardenolides. Insect Biochem Mol Biol 81:51–61 28011348 10.1016/j.ibmb.2016.12.008 PMC5428987

[CR24] Harrington S, Pyche J, Burns AR, Spalholz T, Ryan KT, Baker RJ, Ching J, Rufener L, Lautens M, Kulke D, Vernudachi A, Zamanian M, Deuther-Conrad W, Brust P, Roy PJ (2023) Nemacol is a small molecule inhibitor of C. Elegans vesicular acetylcholine transporter with anthelmintic potential. Nat Comm 14:1816 10.1038/s41467-023-37452-6 PMC10066365 37002199

[CR25] Huynh T, O’Halloran D, Hawdon J, Eleftherianos I (2023) The nematode parasite Steinernema hermaphroditum is pathogenic to Drosophila melanogaster larvae without activating their immune response. microPub Biol 2023:1017912micropubbiology000944 10.17912/micropub.biology.000944 PMC10562934 37822685

[CR26] Karageorgi M, Groen SC, Sumbul F, Pelaez JN, Verster KI, Aguilar JM, Hastings AP, Bernstein SL, Matsunaga T, Astourian M, Guerra G, Rico F, Dobler S, Agrawal AA, Whiteman NK (2019) Genome editing retraces the evolution of toxin resistance in the monarch butterfly. Nature 574:409–412 31578524 10.1038/s41586-019-1610-8 PMC7039281

[CR27] Liu W, Jones AL, Gosse HN, Lawrence KS, Park SW (2019) Validation of the chemotaxis of plant parasitic nematodes toward host root exudates. J Nematol 51:e2019–e2063 34179810 10.21307/jofnem-2019-063 PMC6909389

[CR28] Lohr JN, Meinzer F, Dalla S, Romey-Glusing R, Dobler S (2017) The function and evolutionary significance of a triplicated Na,K-ATPase gene in a toxin-specialized insect. BMC Evol Biol 17:256 29246105 10.1186/s12862-017-1097-6 PMC5732401

[CR29] McGruddy RA, Howse MWF, Haywood J, Ward CJI, Staufer TB, Hayek-Williams M, Toft RJ, Lester PJ (2021) Invasive paper wasps have strong cascading effects on the host plant of monarch butterflies. Ecol Entomol 46:459–469

[CR30] McMullen Ii, J. G., and S. P. Stock. 2014. *In vivo* and *In vitro* Rearing of Entomopathogenic Nematodes (*Steinernematidae*and *Heterorhabditidae*). Journal of Visualized Experiments 10.3791/52096 PMC4828111 25285597

[CR31] Mohammadi S, Yang L, Bulbert M, Rowland HM (2022) Defence mitigation by predators of chemically defended prey integrated over the predation sequence and across biological levels with a focus on cardiotonic steroids. R Soc Open Sci 9:220363 36133149 10.1098/rsos.220363 PMC9449480

[CR32] Peña JM, Carrillo MA, Hallem EA (2015) Variation in the susceptibility of Drosophila to different entomopathogenic nematodes. Infect Immun 83:1130–1138 25561714 10.1128/IAI.02740-14 PMC4333445

[CR33] Peters A (1996) The Natural Host Range of Steinernema and Heterorhabditis spp. and their impact on insect populations. Biocontrol Sci Technol 6:389–402

[CR34] Peterson MA, Dobler S, Holland J, Tantalo L, Locke S (2001) Behavioral, molecular, and morphological evidence for a hybrid zone between Chrysochus auratus and C. cobaltinus (Coleoptera: Chrysomelidae). Ann Ent Soc Amer 94:1–9

[CR35] Rasmann S, Agrawal AA (2011) Latitudinal patterns in plant defense: evolution of cardenolides, their toxicity and induction following herbivory. Ecol Lett 14:476–483 21371232 10.1111/j.1461-0248.2011.01609.x

[CR36] Rasmann S, Kollner TG, Degenhardt J, Hiltpold I, Toepfer S, Kuhlmann U, Gershenzon J, Turlings TC (2005) Recruitment of entomopathogenic nematodes by insect-damaged maize roots. Nature 434:732–737 15815622 10.1038/nature03451

[CR37] Wu SY, Duncan LW (2020) Recruitment of an insect and its nematode natural enemy by olfactory cues from a saprophytic fungus. Soil Biol Biochem 144:107781

[CR38] Yang L, Ravikanthachari N, Marino-Perez R, Deshmukh R, Wu M, Rosenstein A, Kunte K, Song H, Andolfatto P (2019) Predictability in the evolution of Orthopteran cardenolide insensitivity. Philos Trans R Soc Lond B Biol Sci 374:20180246 31154978 10.1098/rstb.2018.0246 PMC6560278

[CR40] Zhang X, van Doan C, Arce CCM, Hu L, Gruenig S, Parisod C, Hibbard BE, Herve MR, Nielson C, Robert CAM, Machado RAR, Erb M (2019) Plant defense resistance in natural enemies of a specialist insect herbivore. Proc Natl Acad Sci U S A 116:23174–23181 31659056 10.1073/pnas.1912599116 PMC6859362

[CR39] Zhang X, Li L, Kesner L, Robert CAM (2021) Chemical host-seeking cues of entomopathogenic nematodes. Curr Opin Insect Sci 44:72–81 33866041 10.1016/j.cois.2021.03.011

[CR41] Zhen Y, Aardema ML, Medina EM, Schumer M, Andolfatto P (2012) Parallel molecular evolution in an herbivore community. Science 337:1634–1637 23019645 10.1126/science.1226630 PMC3770729

