# Supplementary material for: Entomopathogenic Nematode Species Vary in Their Behavior and Virulence in Response to Cardiac Glycosides Within and Around Insect Hosts
Source: J Chem Ecol. 2025 Jan 27;51(1):12. doi: 10.1007/s10886-025-01563-9 (PMC11772503; doi:10.1007/s10886-025-01563-9)
Supplement: Supplementary file 1 — Supplementary Material 1 [file 10886_2025_1563_MOESM1_ESM.docx]

**Supplemental Figures**


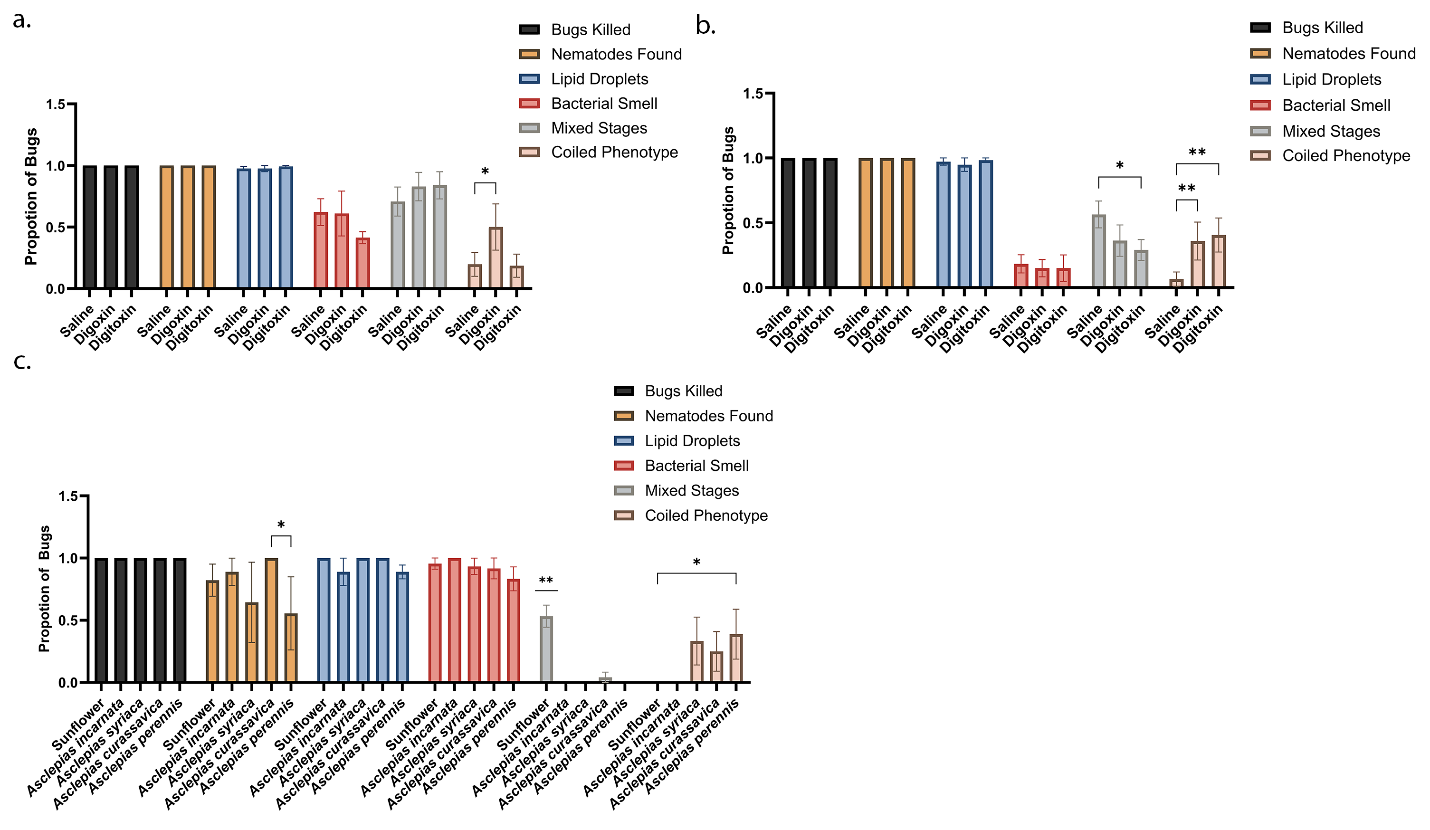


**Supplementary Fig. 1** *Steinernema carpocapsae* shows a better infection rate of large milkweed bugs than *S. feltiae* as observed by detection of odors released by bacterial symbionts. **a-b.** Proportion of large adult milkweed bugs for which phenotypes of interest were observed during dissections of bugs after injection with saline, digoxin or digitoxin, and subsequent infection with *S. carpocapsae* (**a**) and *S. feltiae* (**b**). **c.** Proportion of large adult milkweed bugs for which phenotypes of interest were observed during dissections of bugs reared on different seed diets and after infection with *S. carpocapsae.* Data are represented as mean ± SEM. Statistical analysis was performed using a two-way ANOVA followed by post-hoc Tukey’s analysis.


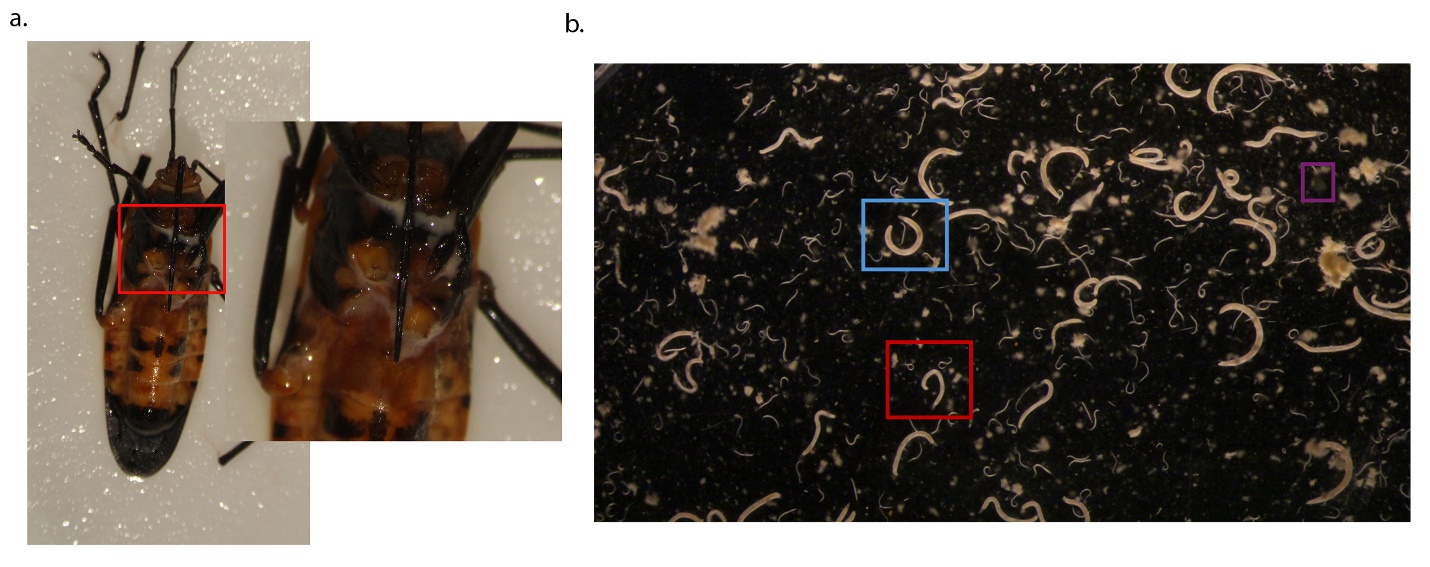


**Supplementary Fig. 2** A successful infection of an *Oncopeltus fasciatus* adult with apparent phenotypes. **a**. Image of an adult large milkweed bug infected by *Steinernema carpocapsae*. **b**. Image of a large milkweed bug dissection upon infection with *S. feltiae.* The blue outline represents a coiling phenotype. The red outline shows mixed stages of the EPN. The purple outline shows a lipid droplet.


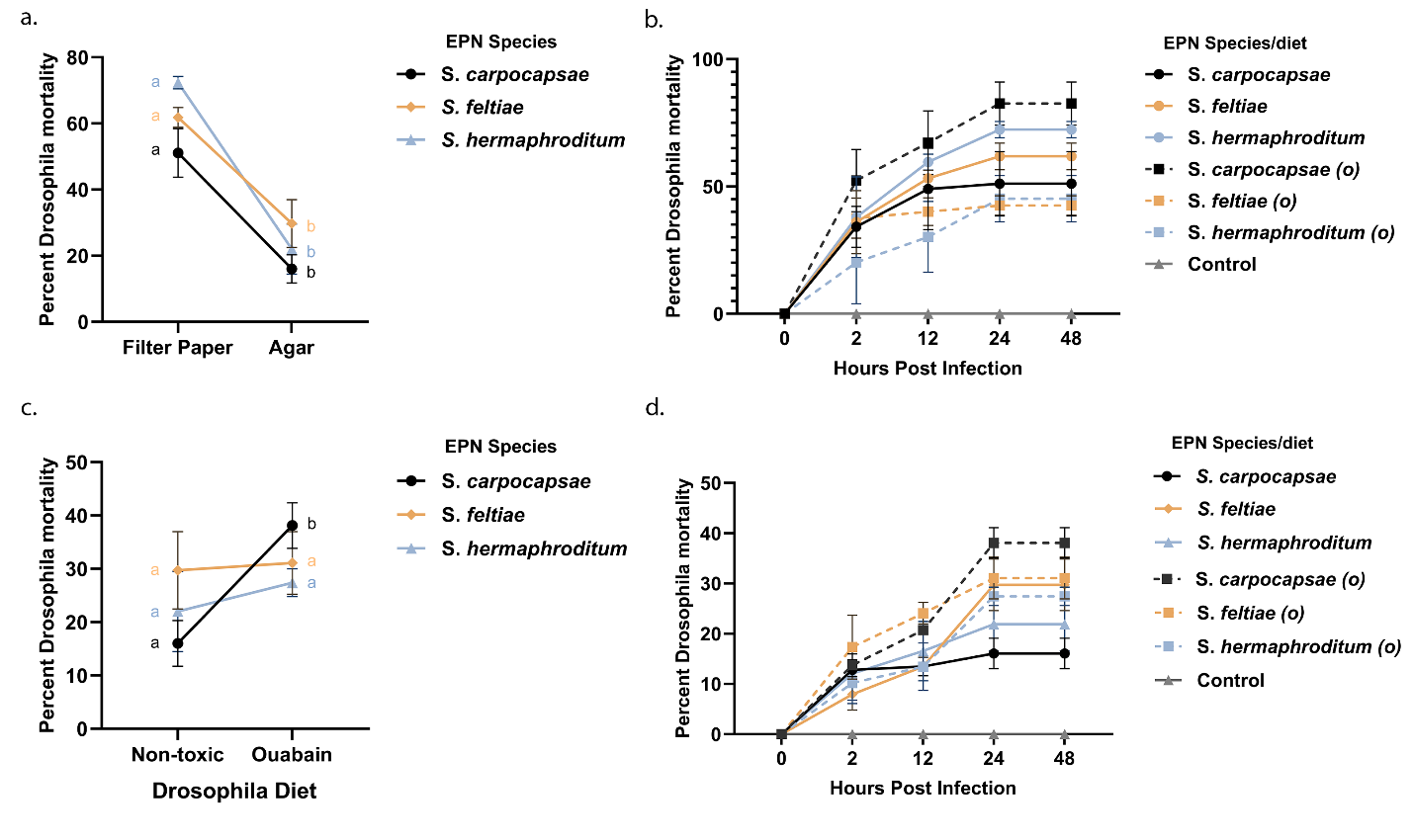


**Supplementary Fig. 3** *Steinernema carpocapsae* shows a significant increase in infections of CG-resistant Drosophila mutants reared on ouabain-containing agar plates. **a.** Percent mortality of mutant Drosophila larvae at 48 hours post infection with the EPN species *S. carpocapsae, S. feltiae,* and *S. hermaphroditum* on agar plates compared to filter paper. **b.** Percent mortality of mutant Drosophila larvae at 2, 12, 24, and 48 hours post infection with *S. carpocapsae, S. feltiae,* and *S. hermaphroditum* on filter paper*.* Fly larvae were fed a regular diet (circles and solid lines) or a diet containing ouabain (squares and dashed lines). The control consists of larvae without EPN infection. **c.** Percent mortality of mutant Drosophila larvae fed on a non-toxic or ouabain diet at 48 hours post infection with the EPN species *S. carpocapsae, S. feltiae,* and *S. hermaphroditum* on agar plates. **d.** Percent mortality of mutant Drosophila larvae at 2, 12, 24, and 48 hours post infection with *S. carpocapsae, S. feltiae,* and *S. hermaphroditum* on filter paper*.* Fly larvae were reared on a non-toxic diet (circles and solid lines) or on an ouabain-containing diet (squares and dashed lines). The control consists of larvae not undergoing an EPN infection. Data are represented as mean ± SEM. Statistical analysis was performed using two-way ANOVA followed by post-hoc Tukey’s analysis.


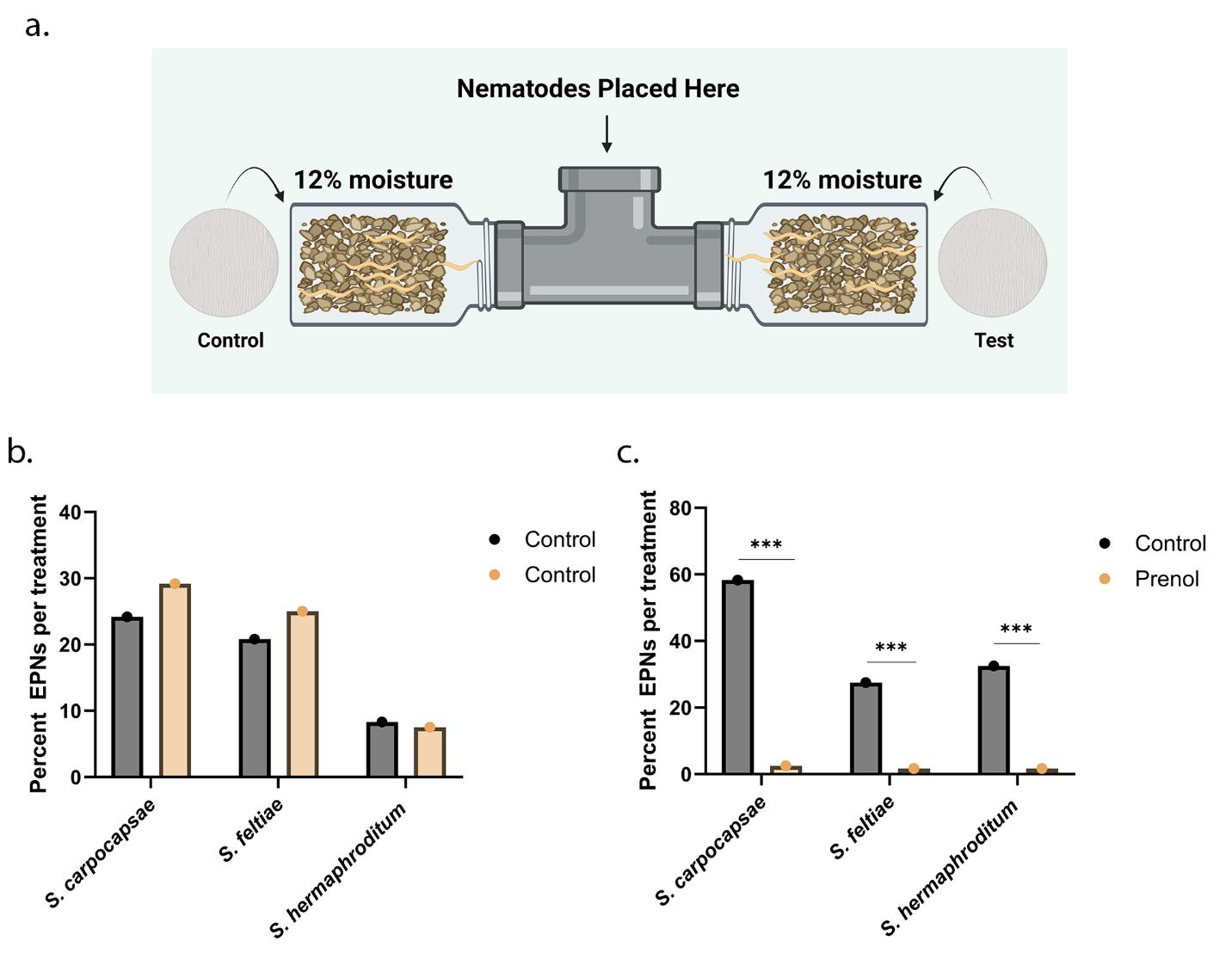


**Supplementary Fig. 4** EPNs behave as expected in negative and positive control experiments in sand. **a.** Setup of the chemotaxis preference assay in sand to test different solutions. **b.** Choice percentages of *Steinernema carpocapsae, S. feltiae,* and *S. hermaphroditum* in chemotaxis assays with tap water as a control. **c.** Choice percentages of *S. carpocapsae, S. feltiae,* and *S. hermaphroditum* in chemotaxis assays with prenol as a negative control for repellence. Data are represented as mean percentages of a trial with three technical replicates for each comparison. Statistical analysis was performed using chi-squared tests on choice values from each individual trial with the control treatment providing the expected values and the test treatments providing the observed values.

**
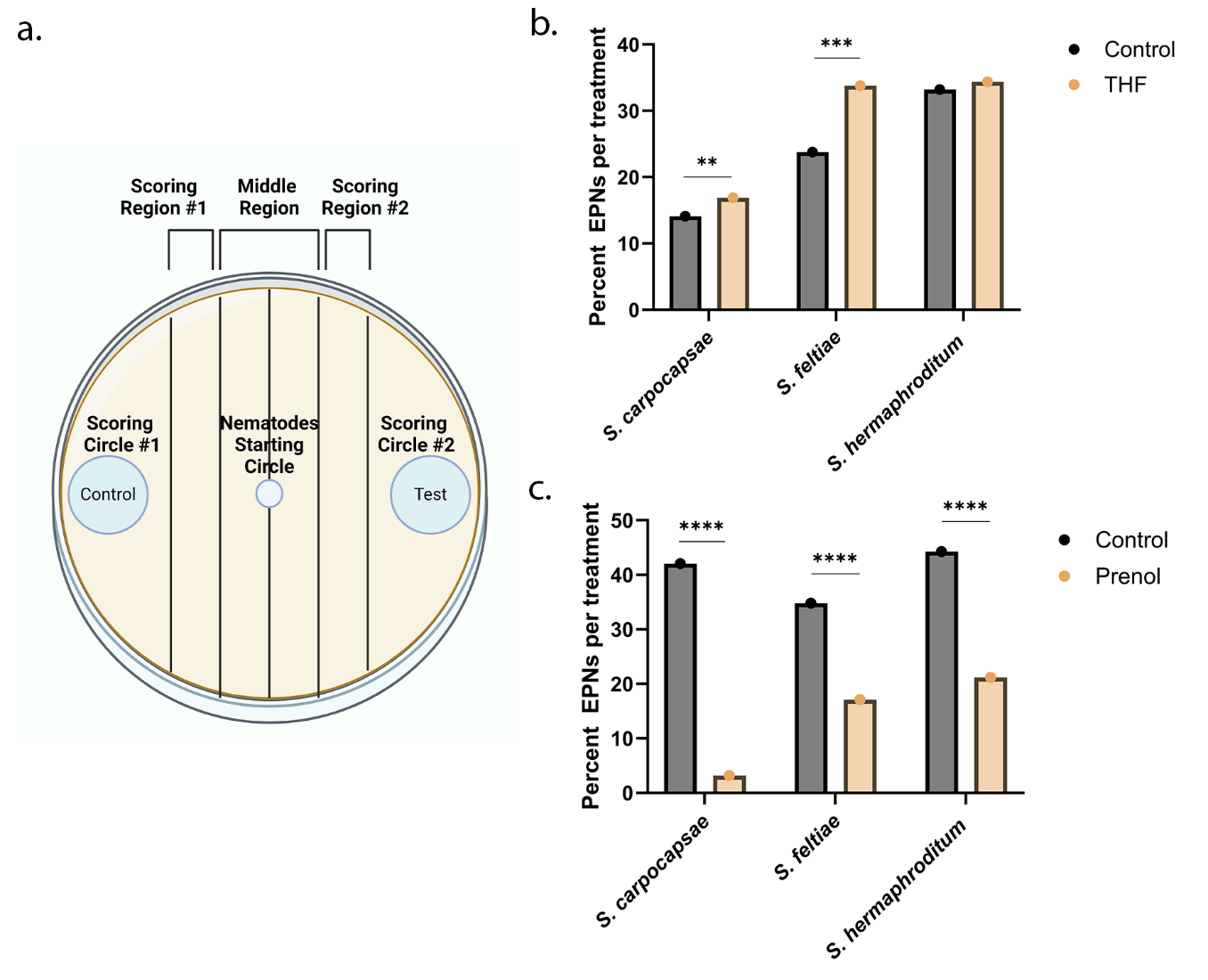
**

**Supplementary Fig. 5** EPNs behave as expected in positive and negative control experiments on agar plates. **a.** Setup of the chemotaxis assay on agar plates. Nematodes were placed in the starting circle and chemical solutions in the scoring circles. **b.** Choice of *Steinernema carpocapsae, S. feltiae,* and *S. hermaphroditum* in chemotaxis assays with the odorant tetrahydrofuran (THF) as a positive control for attraction. **c.** Choice of *S. carpocapsae, S. feltiae,* and *S. hermaphroditum* in chemotaxis assays with the odorant prenol as a negative control for repellence. Data are represented as mean percentages of a trial with three technical replicates for each comparison. Statistical analysis was performed using chi-squared tests on choice values from individual replicates with the control treatment providing the expected values and the test treatments providing the observed values.
